# New *Australovenator* Hind Limb Elements Pertaining to the Holotype Reveal the Most Complete Neovenatorid Leg

**DOI:** 10.1371/journal.pone.0068649

**Published:** 2013-07-24

**Authors:** Matt A. White, Roger B. J. Benson, Travis R. Tischler, Scott A. Hocknull, Alex G. Cook, David G. Barnes, Stephen F. Poropat, Sarah J. Wooldridge, Trish Sloan, George H. K. Sinapius, David A. Elliott

**Affiliations:** 1 School of Engineering, The University of Newcastle, Callaghan, New South Wales, Australia; 2 Australian Age of Dinosaurs Museum of Natural History, Winton, Queensland, Australia; 3 Department of Earth Sciences, University of Oxford, Oxford, United Kingdom; 4 Ancient environments, Queensland Museum, Hendra, Queensland, Australia; 5 Monash Biomedical Imaging, Monash University, Clayton, Victoria, Australia; 6 Monash e-Research Centre, Monash University, Clayton, Victoria, Australia; 7 Life Sciences Computation Centre, Parkville, Victoria, Australia; 8 Department of Earth Sciences, Uppsala University, Villavägen, Uppsala, Sweden; 9 Queensland Xray, Mackay, Queensland, Australia; University of Pennsylvania, United States of America

## Abstract

We report new skeletal elements pertaining to the same individual which represents the holotype of *Australovenator wintonensis*, from the ‘Matilda Site’ in the Winton Formation (Upper Cretaceous) of western Queensland. The discovery of these new elements means that the hind limb of *Australovenator* is now the most completely understood hind limb among Neovenatoridae. The new hind limb elements include: the left fibula; left metatarsal IV; left pedal phalanges I-2, II-1, III-4, IV-2, IV-3; and right pedal phalanges, II-2 and III-1. The detailed descriptions are supported with three dimensional figures. These coupled with the completeness of the hind limb will increase the utility of *Australovenator* in comparisons with less complete neovenatorid genera. These specimens and the previously described hind limb elements of *Australovenator* are compared with other theropods classified as neovenatorids (including *Neovenator*, *Chilantaisaurus*, *Fukuiraptor, Orkoraptor* and *Megaraptor*). Hind limb length proportion comparisons indicate that the smaller neovenatorids *Australovenator* and *Fukuiraptor* possess more elongate and gracile hind limb elements than the larger *Neovenator* and *Chilantaisaurus.* Greater stride lengths to body size exist in both *Fukuiraptor* and *Australovenator* with the femur discovered to be proportionally shorter the rest of the hind limb length. Additionally *Australovenator* is identified as possessing the most elongate metatarsus. The metatarsus morphology varies with body size. The larger neoventorids possess a metatarsus with greater width but shorter length compared to smaller forms.

## Introduction

**Figure 1:**
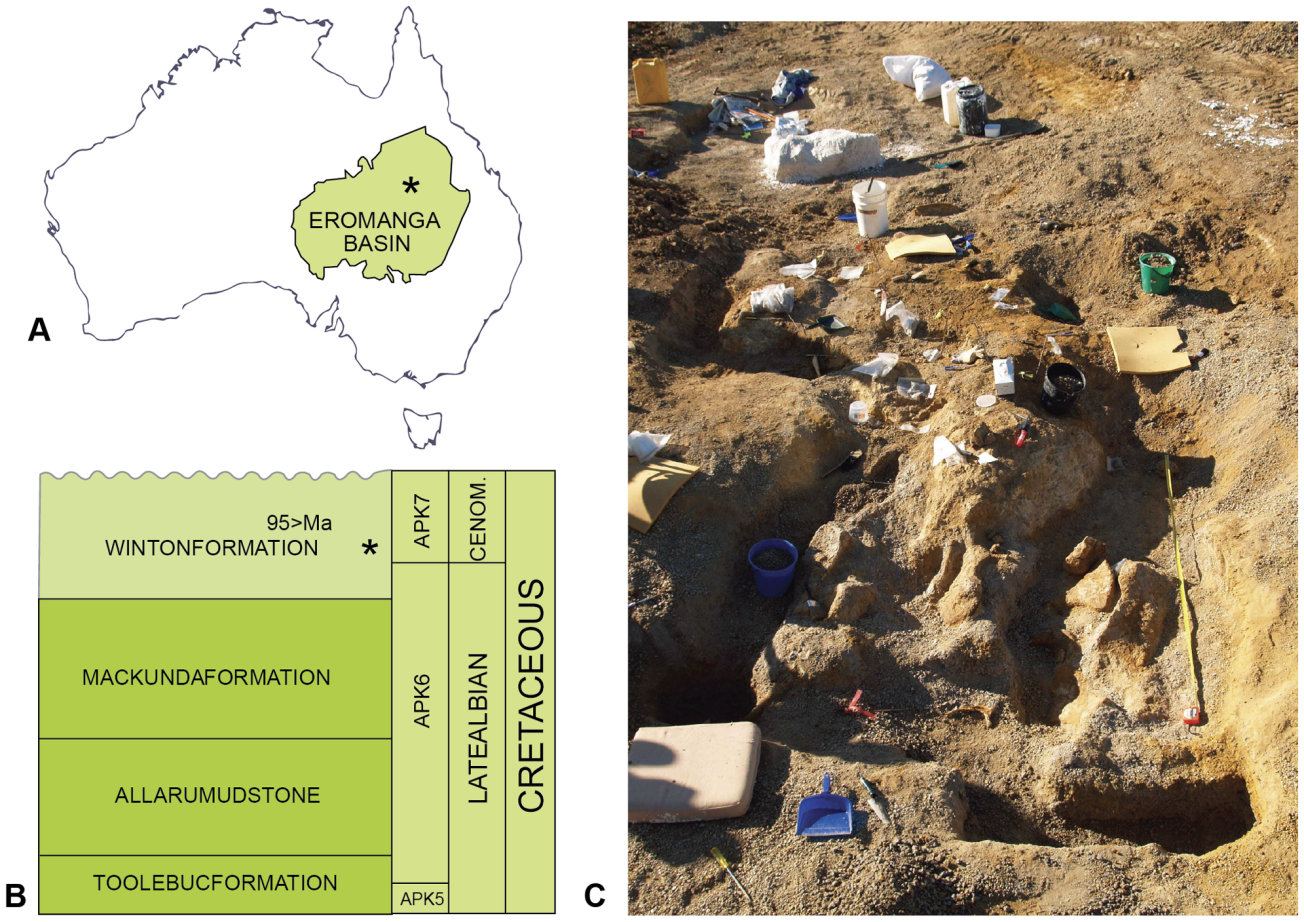
Locality and dig site. Australian Age of Dinosaur locality ‘Matilda site’. The Eromanga basin and site locality (A), formation and age (B), dig site (C).

The skeletal remains of *Australovenator wintonensis* [1] were discovered interspersed with the remains of a sauropod dinosaur *Diamantinasaurus matildae* [1]. The fossils were excavated from Australian Age of Dinosaurs Locality 85 (AODL 85) – the “Matilda site” on Elderslie station, approximately 60km northwest of Winton, Queensland, Australia. Samples from the Matilda underwent zircon dating indicating a Cenomanian age (*ca.* 95 Ma) for the site (Figure 6 in [2]) [2], [3]. The deposit was first identified by the landowners, who discovered large fragmented sauropod remains exposed on the surface. Excavation of the site demonstrated that the bones were being reworked from gunmetal blue-coloured clay, rich in plant debris (Figure 1; Figure S1). The plant material consists of a diverse range of macro and micro fauna flora [4]-[10]. The deposit was interpreted as an abandoned channel fill or oxbow lake [1]. Most of the specimens were found to be encased in a concretionary phosphatic crust. Although substantial skeletal remains of *Austraovenator* were reported in the description of the holotype [1], the preparation of concretions from AODL 85 continued following the publication of the paper, yielding new forelimb [11] and hind limb elements of *Australovenator*.

Herein we describe new hind limb elements pertaining to the holotype individual of *Australovenator wintonensis* (Australian Age of Dinosaur Fossil 604 [AODF 604]). The new hind limb elements described include: the left fibula; left metatarsal IV; left pedal phalanges I-2, II-1, III-3, III-4, IV-2, IV-3; and right pedal phalanges, II-2 and III-1. We also revise the identifications of the previously described pedal phalanges. These corrections were made based on comparisons with phalanges of *Allosaurus fragilis* [12], *Neovenator salerii* [13] and the emu (*Dromaius novaehollandiae*). The pedal phalanges described in the initial description of *Australovenator*, when assigned to their correct positions, represent left pedal phalanx, IV-1 and right pedal phalanges I-2, II-3, III-2, III-3, IV-4 and IV-5. The near completeness of the hind limb of *Australovenator* will increase its utility in comparisons with less complete neovenatorid genera, particularly by ensuring that their pedal element will be interpreted correctly.

## Methods

### Fossil Preparation

Specimens were prepared using pneumatic air scribes and chisels. They were consolidated with Paraloid B72. Polyethylene Glycol' PEG 3350 ‘Carbowax’ was used to support fragile fossil specimens during preparation, filling in gaps and cracks for extra support and absorbing vibration caused by the pneumatic preparation tools.

### Specimens

All necessary permits were obtained for the described study, which complied with all relevant regulations. Permission to excavate the specimens from Elderslie station was obtained from the landholders. During excavation each specimen is given a preliminary field number for location and storing purposes. Once the specimens have been prepared and formally identified they are donated by the landholder to the Australian Age of Dinosaur Museum of Natural History (AAOD). All specimens pertaining to the holotype *Australovenator wintonensis* are allocated the specimen number AODF604. The specimens are stored in a climate controlled type room at the Australian Age of Dinosaurs Museum 15km east of Winton, Queensland, Australia.

### Computed Tomography

The *Australovenator* specimens were computed tomography (CT) scanned at Queensland Xray, Mackay Mater Hospital, central eastern Queensland using a Philips Brilliance CT 64-slice machine which produced 0.9mm slices. Mimics® version 10.01 software, was used to view internal structures in cross-section and to create three dimensional renders. These were subsequently scanned to obtain an external mesh. The meshes were then imported into the graphic design package Rhinoceros® 4.0, which was used to develop rendered meshes of fossil specimens enabling the morphology to be clearly viewed alongside actual specimens.

### 3-d Figures

Individual meshes of fossil specimens were loaded into a custom program that loads an Alias Wavefront (.obj)-format mesh and compresses it into the Product Representation Compact (PRC)-format (International Organization for Standardization Draft International Standard ISO/DIS 14739-1.3), suitable for embedding in a Portable Document Format (PDF) file as an interactive, 3-dimensional figure. We used a modified version of the program xrw2pdf from the S2VOLSURF tools [14], based on the S2PLOT programming library [15], [16].

PRC files were embedded in PDF documents as interactive figures using the LaTeX document preparation system, the movie15 style file for LaTex supporting multimedia enhancements to PDF documents, and the JavaScript file s2plot-prc.js included with S2PLOT. When viewed in Adobe Reader or Adobe Acrobat on desktop systems (Microsoft Windows, Apple Macintosh OS X, Linux), the resultant supplementary 3-d figures enable the interactive rotation, zooming, and relighting of the fossil meshes.

## Results and Discussion

The new hind limb and pedal elements described below were initially identified by comparison with *Allosaurus fragilis* (Plates 53–55 in [17]) and *Neovenator salerii* (Figure 25 and Plates 44–45 in [18]). The preservation of *Australovenator* phalanges enabled rearticulation of adjacent elements. We present detailed figures and supplementary three dimensional PDFs of the specimens to support our morphological descriptions.

**Figure 2:**
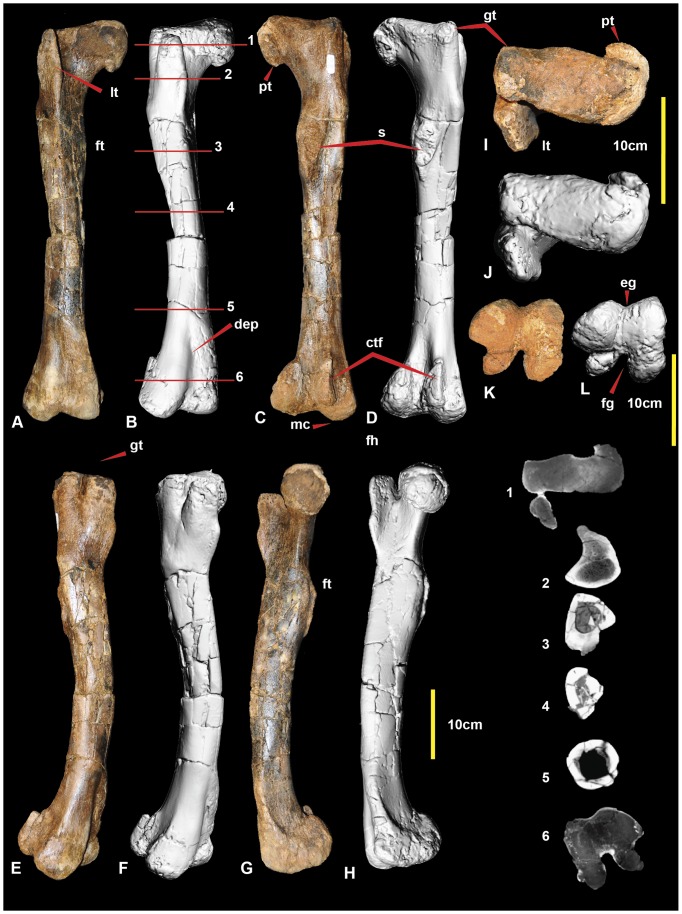
Right femur. Right femur in: cranial (A & B); caudal (C & D); lateral (E & F); medial (G & H); proximal (I & J); and distal (K & L) views. Abbreviations: ctf, crista tibiofibularis; dep, depression; eg, extensor groove; fg, flexor groove; ft, fourth trochanter; gt, greater trochanter; lt, lesser trochanter; mc, medial condyle; cf, caudal flange on caput; s, scar; 1-6 CT sections.

**Figure 3:**
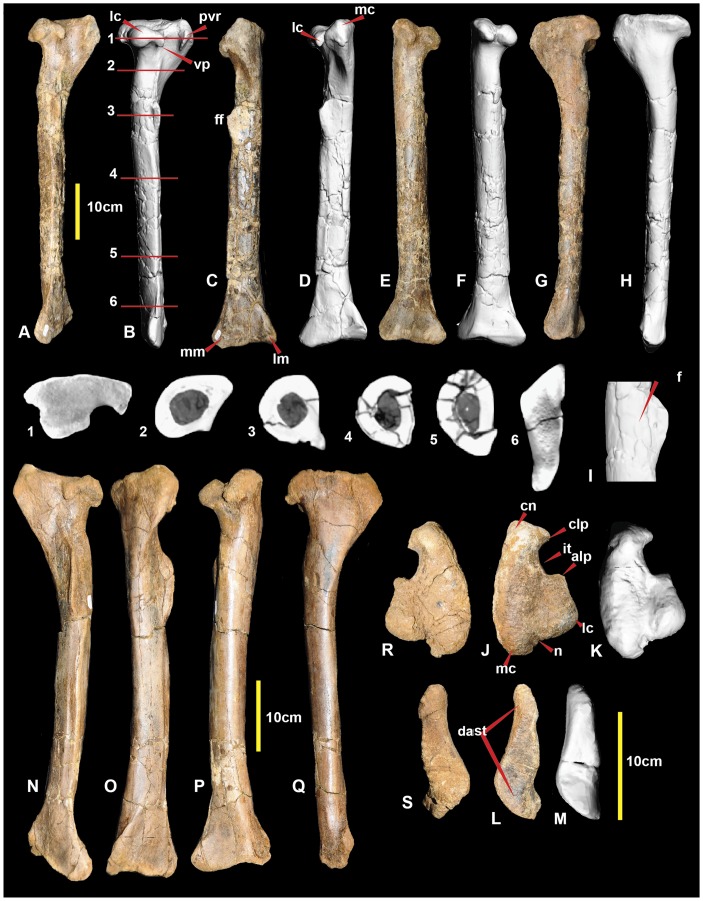
Right and left tibia. Right tibia in: lateral (A & B); cranial (C & D); caudal (E & F); and medial (G & H) views; close up of fibular flange with fossa (I), right tibia in: proximal (J & K); and distal (L & M) views. Left tibia in: lateral (N); cranial (O); caudal (P); medial (Q); proximal (R); and distal (S) views. Abbreviations: alp, anterolateral process; clp, cranio-lateral process; cn, cnemial crest; dast, distal astragular facet; f, fossa; ff, fibular flange; it, incisura tibialis; lc, lateral condyle; lm, lateral malleolus; mc, medial condyle; mm, medial malleolus; n, notch; pvr, postero-ventral ridge; vp, ventral spine-like process.

**Figure 4:**
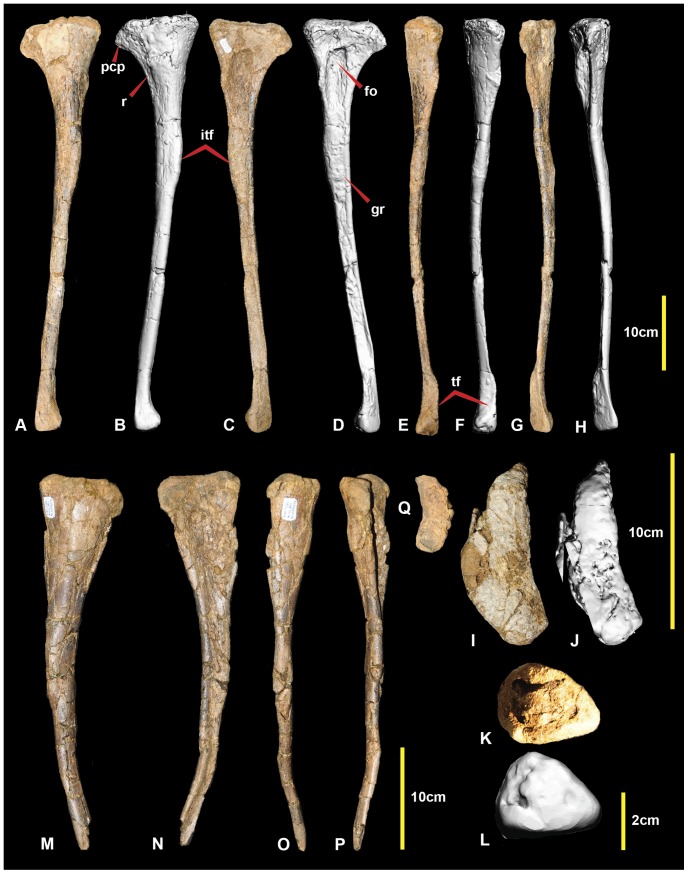
Right and left fibula. Right fibula in: medial (A & B); lateral (C & D); caudal (E & F); cranial (G & H); proximal (I & J); and distal (K & L) views. Left fibula in: medial (M); lateral (N); caudal (O); cranial (P); and proximal (Q) views. Abbreviations: fo, fossa; gr, groove; itf, attachment area for interosseum tibiofibulare ligaments; pcp, proximo-cranial process; r, ridge; tf, tibial facet.

**Figure 5:**
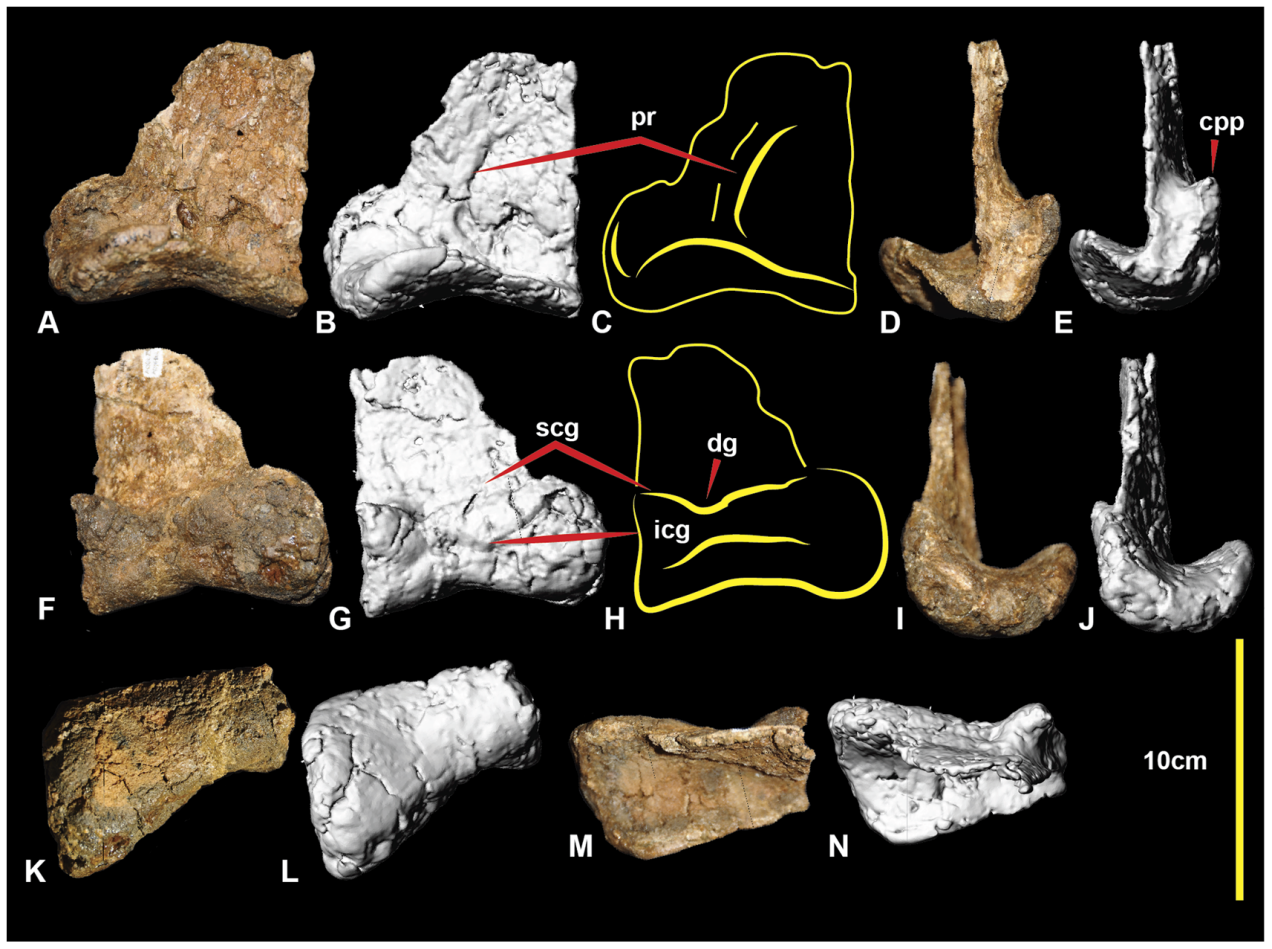
Right astragalus. Right astragalus in: caudal (A, B & C); lateral (D & E); cranial (F, G, H); medial (I & J); distal (K & L); and proximal (M & N) views. Abbreviations: cpp, cranio-proximal process; dg, distal groove; icg, inferior cranial groove; scg, superior cranial groove; pr, caudal ridge.

**Figure 6:**
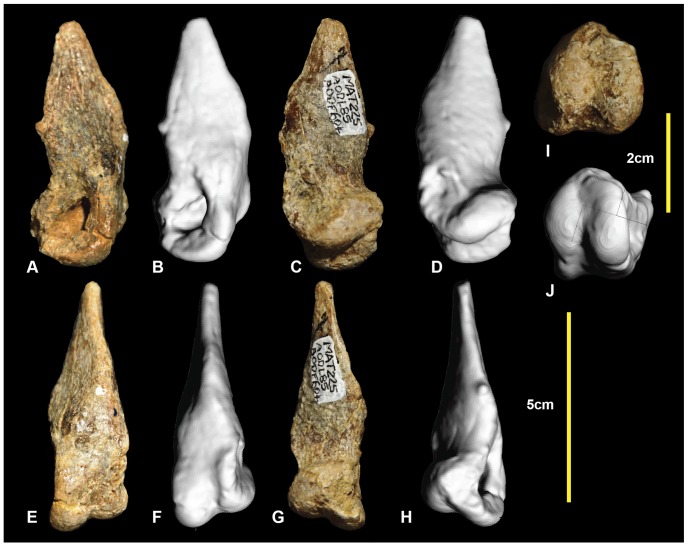
Left metatarsal I. Left metatarsal I in: lateral (A & B); medial (C & D); ventral (E & F); dorsal (G & H); and distal (I & J) views.

**Figure 7:**
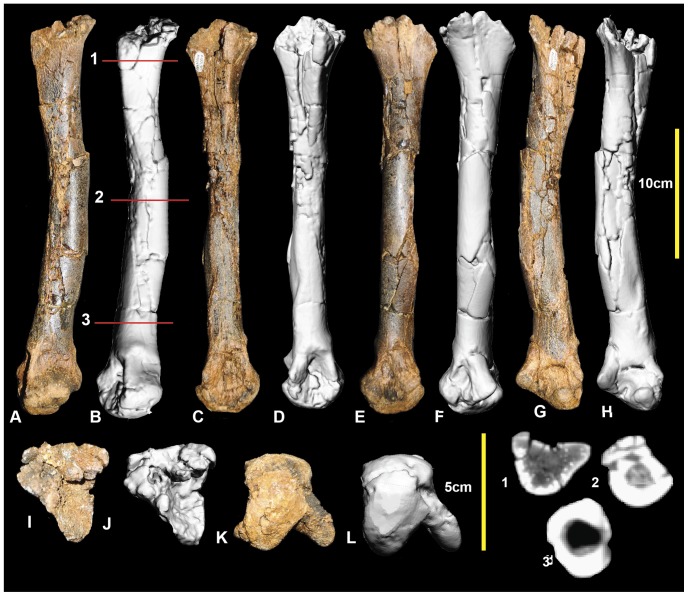
Right metatarsal II. Right metatarsal II in: dorsal (A & B); lateral (C & D); medial (E & F); ventral (G & H); proximal (I & J); and distal (K & L) views; and metatarsal II cross-sections (1–3).

**Figure 8:**
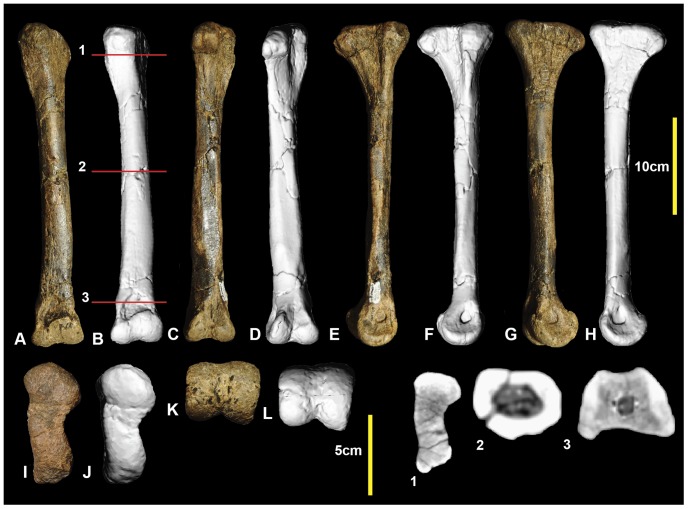
Right metatarsal III. Right metatarsal III in: dorsal (A & B); ventral (C & D); lateral (E & F); medial (G & H); proximal (I & J); and distal (K & L) views; and and metatarsal III cross-sections (1–5).

The following specimens were described in the initial description of *Australovenator wintonensis* [1]: the right femur (Figure 2; Figure S2), right and left tibiae (Figure 3; Figure S3), right fibula (Figure 4; Figure S4), right astragalus (Figure 5; Figure S5), left metatarsal I (Figure 6; Figure S6), and right metatarsals II (Figure 7; Figure S7) and III (Figure 8; Figure S8). Left metatarsal I was originally identified as a right element [1]. These specimens, or their counterparts from the limb of the opposite side, were adequately described [1]. Four pedal phalanges were figured and described as part of the original description [1]. Their descriptions have been revised as their correct positions are now known.

### Left Fibula (Figure 4)

The original description of the right fibula (Figure 4A–L) was adequate [1]. The left fibula, reported here (Figure 4M–Q), is missing its distal portion: however, the proximal end is complete, unlike the left fibula, revealing a proximally flatter and more rounded lateral surface. Post-mortem distortion has morphed the proximomedial fossa so that it is more ovoid than in the right fibula and has also caused the shaft to be bent distally. Measurements are given in Table 1.

**Table 1:** Femur, tibia, fibula, metatarsal, and astragalar measurements of *Australovenator wintonensis*.

	Proximal width from greater trochanter to head	Shaft length	Mid-shaft width	Mid-shaft height	Mid shaft circumference	Distal height medial-lateral	Distal width	Proximal width	Proximal height
Right Femur	132	578	75	80	179	100	120		
Right Tibia		564	45	54	168	40	123	140	
Left Tibia		569			152		138	135	
Left Fibula		538	59		66		350	95	40
Fibula		538*			61	NP	NP	110	
Left MTI		66			67 64	24	20		
Right MTII		284	26	27	91	42, 40	46	48*	53*
Right MTIII		322	29	23	93	48, 42	51	40	74
Left MTIV		272, 300*	34	21	97	NP	NP	522	62

Right Astragalus	Ascending process height	Condylar width	Total height	Cranio-caudal length of medial condyle					
	74	105	102	64					

Hind limb measurements which include the specimen lengths and estimates where the specimen is not entirely preserved are marked with an asterisk (*).

### Left metatarsal IV (Figure 9; Figure S9)

Metatarsal IV was poorly preserved, with the shaft sustaining multiple post-mortem fractures. The distal condyle is missing. The shaft is concave medially at the proximal end of the shaft forming an articular surface for metatarsal III. The lateral surface is slightly concave for its articulation with metatarsal V. Disto-laterally there is a depression just proximal to the lateral condyle. The shaft is elongate and approximately straight, as in metatarsals III and II. The cross-section of the mid-shaft is crescentic for most of its length (Figure 9.2), with the lateral face slightly concave and medial face convex. A distal cross-sectional is oval with very thick cortical bone and a narrow ovoid medullary cavity. The lateral face becomes convex distally, resulting in a rounded cross-section (Figure 9.3). Although the distal condyles are missing, their proximally preserved portion suggests that the lateral condyle was ventrolaterally angled, and was taller than the medial condyle which is transversely broad. Measurements are given in Table 1.

**Figure 9:**
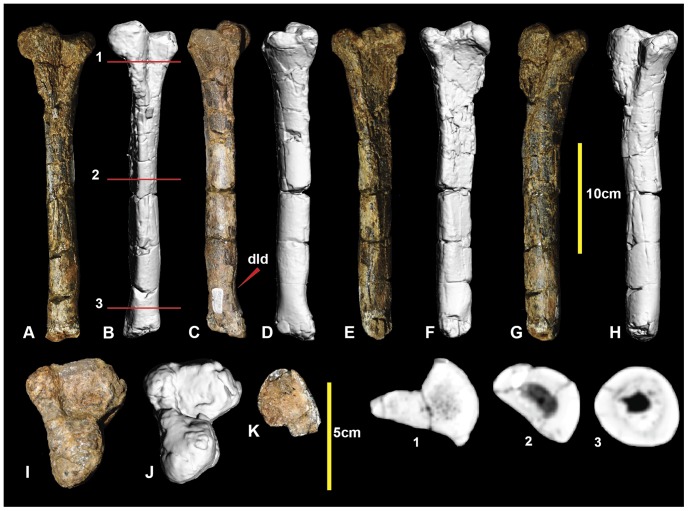
Left metatarsal IV. Left metatarsal IV in: ventral (A & B); dorsal (C & D); lateral (E & F); medial (G & H); proximal (I & J); and distal (K) views; and metatarsal IV cross-sections (1–3).

### Right metatarsus (Figure 10; Figure S10)

Metatarsals I and IV from the left foot have been mirrored to reconstruct an articulated metatarsus. Metatarsal II was digitally straightened as the specimen was deformed with an unnatural medial bend. Despite this deformation, both metatarsals II and III have a distinct articulation both proximally and distally. The mirrored metatarsal IV articulates well at the proximal end. The metatarsus is quite gracile relative to the more robustly built metatarsi of the less derived *Neovenator*, *Chilantaisaurus* and *Allosaurus*. The general morphology of the metatarsus is that of an undrived theropod metatarsi, where the proximal shaft of metatarsal III is visible in cranial and caudal views and the distal portion of the shaft is circular which is distinctly different to the morphological features of an arctometatarsus or subarctometatarsus (Figure 1 in [19]).

**Figure 10:**
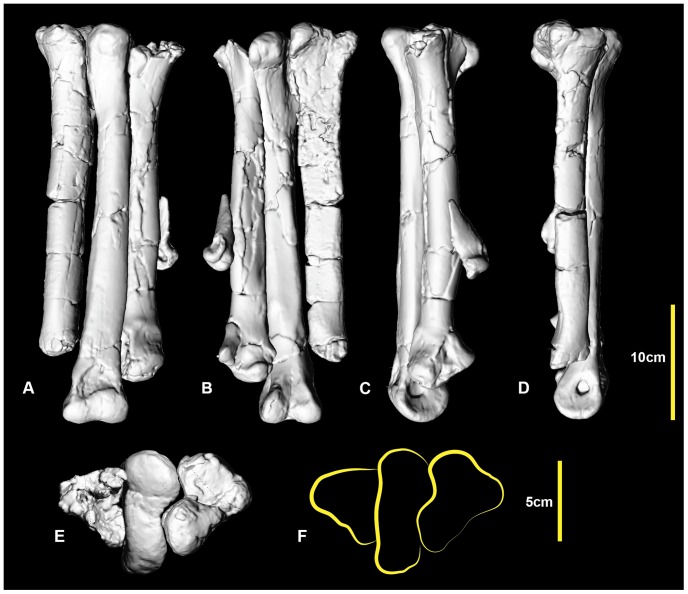
Reconstructed right metatarsus. Reconstructed right metatarsus in: dorsal (A); ventral (B); medial (C); lateral (D); and proximal (E, F) views.

Measurements are given in Table 2.

**Table 2:** Hind limb lengths and hind limb element ratios.

Dinosaur	Femur length	Tibia length	Metatarsus length	Metatarsus width	THL	FL/THL %	TL/THL	MTL/THL	MTW/THL	MT/FL
***Allosaurus fragilus*** ** UUVP6000r**	880	730	375	130*	1985	44.33%	36.77%	18.90%	6.55%	42.61%
***Chilantaisaurus tashuikouensis*** ** IVPP V.2884.7**	1190	954	450	200*	2594	45.87%	36.77%	17.36%	7.71%	37.81%
***Neovenator salerii***	730	680	340*	110	1750	41.71%	38.85%	19.44%	6.28%	46.57%
***Australovenator wintonensis***	578	564	322	89	1464	39.48%	38.52%	21.99%	6.07%	55.7%
***Fukuiraptor kitadaniensis***	507	∼507	297.5	un	1311	38.67%	38.67%	22.69%	un	58.67%

Measurements in this table were obtained from [17], [18], [25].

Comparisons of hind limb length ratios of allosaurid and neovenatorid theropod dinosaurs. Asterisks (*) mark lengths taken from published illustrations. A length averaged is indicated (∼) due to missing elements. Unknown measurements (un). Measurements in (mm).

### Left and right pedal phalanx I-2 (Figure 11; Figure S11)

**Figure 11:**
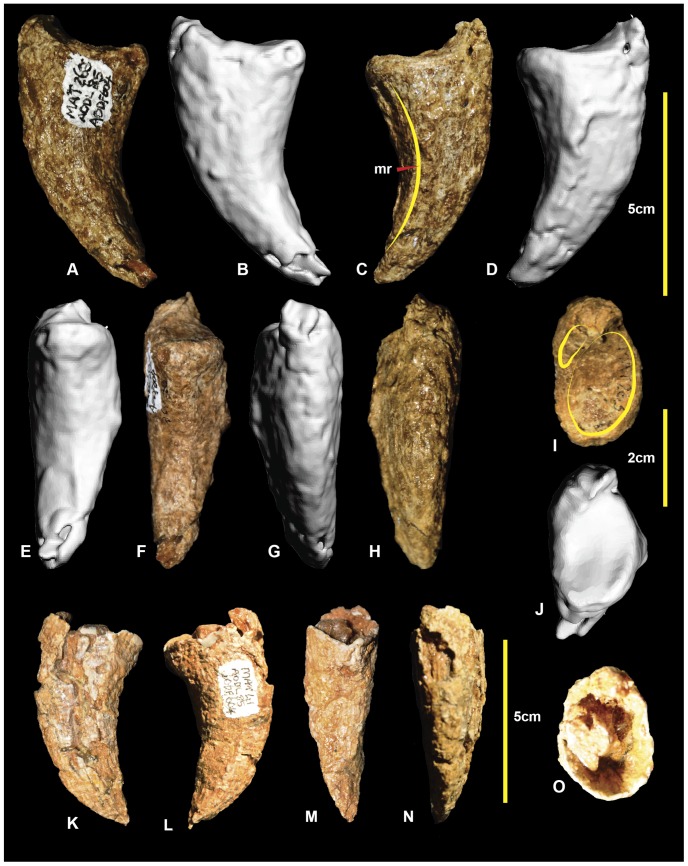
Left and right pedal phalanges I-2. Left pedal phalanx I-2 in: lateral (A & B); medial (C & D); ventral (E & F); dorsal (G & H); and proximal (I & J) views. Right pedal phalanx I-2 in; medial (K); lateral (L); ventral (M); dorsal (N); and proximal (O) views.

The first pedal digit of basal theropods comprises two phalanges. Only the distal (ungual) phalanx, I-2, is known in *Australovenator*. The right pedal phalanx I-2 (Figure 11K-O) was originally mistaken for the distal tip of manual phalanx II-3 [1]. The discovery and description of additional manual elements resulted in its correct identification as a pedal phalanx [11]. The right specimen is missing the articular facet; however, the left is complete. The lateral surface is rounded, whereas the medial surface bears a distinct ventromedial ridge. The proximal articular surface is divided into two articular facets. The medial facet is large and depressed whilst the lateral facet is small and is located dorsomedially. It is similar in morphology to *Allosaurus fragilis* (Plate 54 in [17]). Measurements are given in Table 3.

**Table 3:** Pedal phalanx and ungual measurements of *Australovenator wintonensis*.

Specimens	Proximal width	Proximal height	Shaft length	Medial length	Lateral length	Distal dorsal width	Distal ventral width	Lateral condyle height	Medial condyle height
Left MTI-2	19	28	66						
Left MTII-1	33*	43*	102, 106*	98, 106*	96, 106*	35	43	33	37
Left MTII-2	35*	30*	60, 64*	57, 60*	51, 60*	18, 24*	21,27*	25*	23*
Right MTII-3	25	32	84						
Right MTIII-1	NP	NP	106, 115*			44	48	37	37
Right MTIII-2	46	42		106	10	32	40	32	32
Left and Right MTIII-3	41	34	73	73	73	28	28	26	26
Left MTIII-4	301	36	95						
Left MTIV-1	39	46	82	76	82	37	42	28	36
Left MTIV-2	35	30	49	49	44		33*		
Left MTIV-3	31	29	46	46	46	19	27	23	25
Right MTIV-4	28	26	33	33	32	13	23	20	22
Right MTIV-5	22	27	77						

The pedal phalanx measurements which include the specimen lengths and estimates where the specimen is not entirely preserved are marked with an asterisk (*). Portions not preserved are denoted by NP.

### Left pedal phalanx II-1 (Figure 12; Figure S12)

In contrast with pedal digit I, the second pedal digit is more completely known, with one example of all three phalanges preserved. The phalanx is broken proximally, meaning that the proximal articular surface is missing. The shaft is elongate and curved medially. The mid-shaft cross section is circular (Figure 12.2) becoming trapezoidal distally (Figure 12.3). The medial distal condyle is taller and broader than the lateral condyle. In distal view, the medial condyle appears to be dorsoventrally orientated, whereas the lateral condyle is slanted laterally. Both condyles have very shallow collateral ligament pits. Measurements in Table 3.

**Figure 12:**
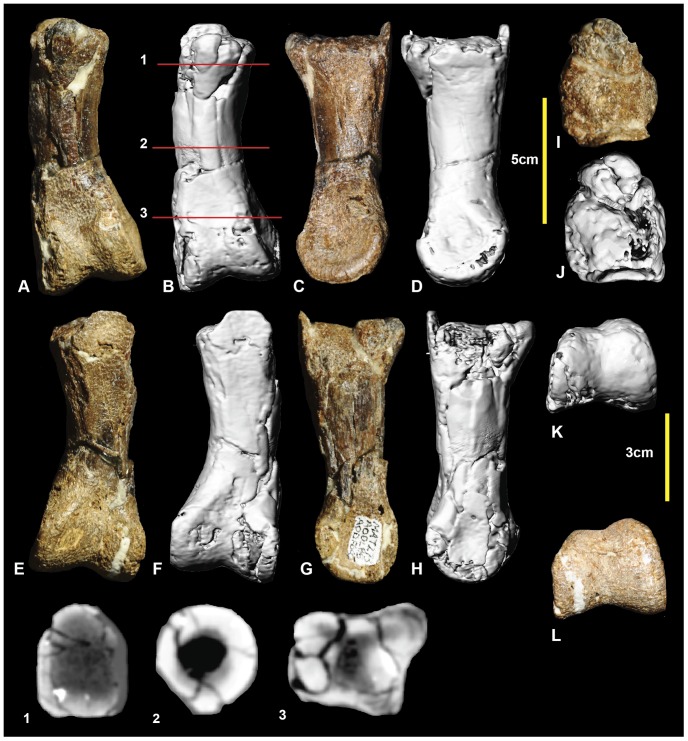
Left pedal phalanx II-1. Left pedal phalanx II-1 in: dorsal (A & B); medial (C & D); ventral (E & F); lateral (G & H); proximal (I & J); and distal (K & L) views; and left pedal phalanx II-1 cross-sections (1–3).

### Left pedal phalanx II-2 (Figure 13; Figure S13)

Pedal phalanx II-2 is poorly preserved, with both the proximal and distal ends incomplete. The specimen is proportionally short compared to II-1, and has a rounded trapezoidal cross-section (Figure 13.1). Although the proximal articular facets are poorly preserved, the lateral appears broader and taller than the medial. At the distal end, both medial and lateral condyles have deep collateral ligament pits. The preserved morphology of the distal condyles suggests that the medial condyle was orientated dorsoventrally and the lateral condyle is angled laterally. A well-defined hyper-extensor pit is present on the dorsal surface immediately proximal to the distal condyles. Measurements in Table 3.

**Figure 13:**
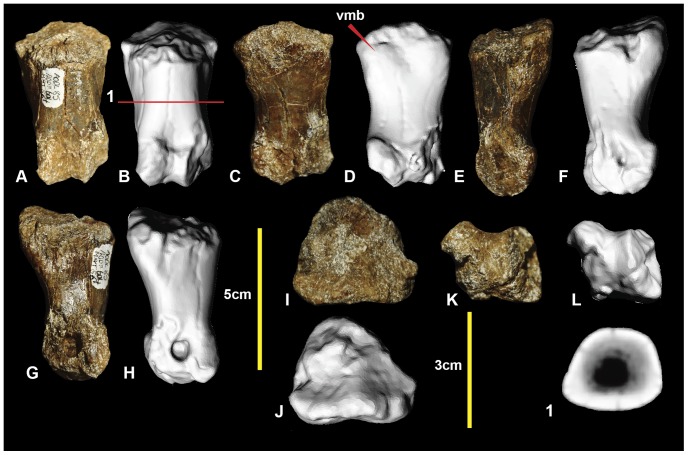
Left pedal phalanx II-2. Left pedal phalanx II-2 in: dorsal (A, B); ventral (C, D); lateral (E, F); medial (G, H); proximal (I, J); and distal (K, L) views; and left pedal phalanx II-2 cross-section (1).

### Right pedal phalanx II-3 (Figure 14; Figure S14)

Pedal phalanx II-3, the ungual of the second digit, is well preserved. It has a subtriangular cross-section (Figure 14.1), is recurved, and tapers distally to a sharp point. The medial and lateral vascular grooves are symmetrical. The proximal articular facet is tall and oval with the medial articular facet being slightly taller than the lateral facet. There is a rounded flexor tubercle on the ventral surface. Measurements in Table 3.

**Figure 14:**
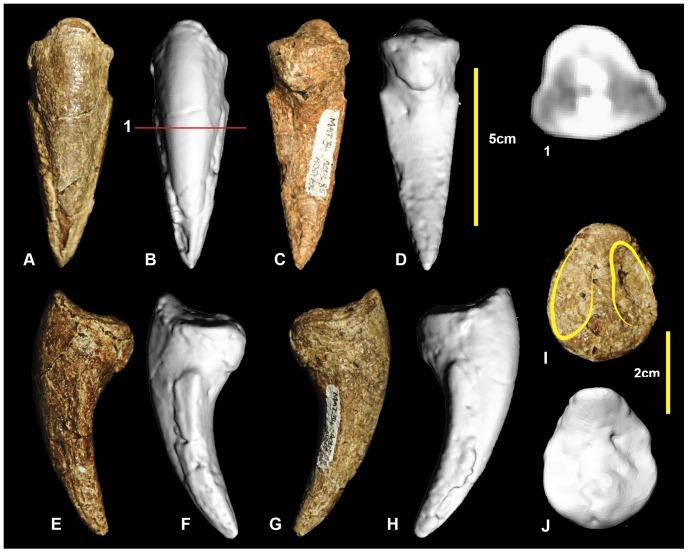
Right pedal phalanx II-3. Right pedal phalanx II-3 in: dorsal (A & B); ventral (C & D); medial (E & F); lateral (G & H); and proximal (I & J) views; and right pedal phalanx II-3 ungual cross-section (1).

### Right pedal phalanx III-1 (Figure 15; Figure S15)

Examples of all four phalanges of the third digit are preserved. Pedal phalanx III-1 is symmetrical along its sagittal plane and is slender at mid-shaft, with pronounced expansion of the distal condyles. The proximal end is poorly preserved with only a small portion of the articular facet preserved. The mid-shaft is circular in cross-section (Figure 15.1). In distal view the medial condyle is slightly taller than the lateral condyle. Both condyles have deep collateral ligament pits. Measurements in Table 3.

**Figure 15:**
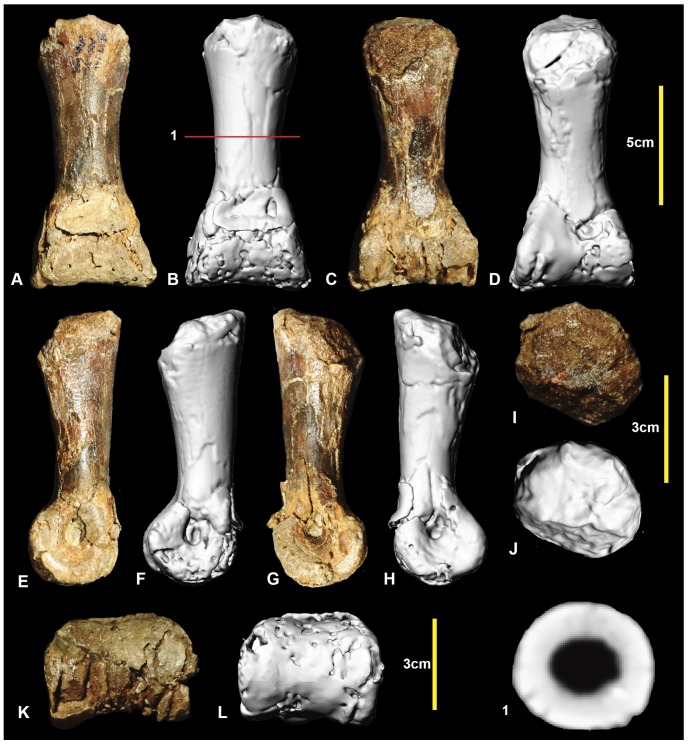
Right pedal phalanx III-1. Right pedal phalanx III-1 in: dorsal (A & B); ventral (C & D); lateral (E & F); medial (G & H); proximal (I & J); and distal (K & L) views; and right pedal phalanx III-1 mid-shaft cross-section (1).

### Right pedal phalanx III-2 (Figure 16; Figure S16)

Pedal phalanx III-2 is complete. It is elongate and nearly symmetrical. The mid-shaft is circular in cross-section (Figure 16.1). A shallow triangular depression is located proximally on the ventral surface of the shaft. The proximal articular facet is slightly taller on the medial side. It is concave and does not possess distinct facets for the medial and lateral condyles of manual phalanx III-1. A well-defined hyperextensor pit is located on the dorsal surface immediately proximal to the distal condyles. The lateral condyle is slightly taller than the medial condyle. Both medial and lateral distal condyles have deep collateral ligament pits. Measurements in Table 3.

**Figure 16:**
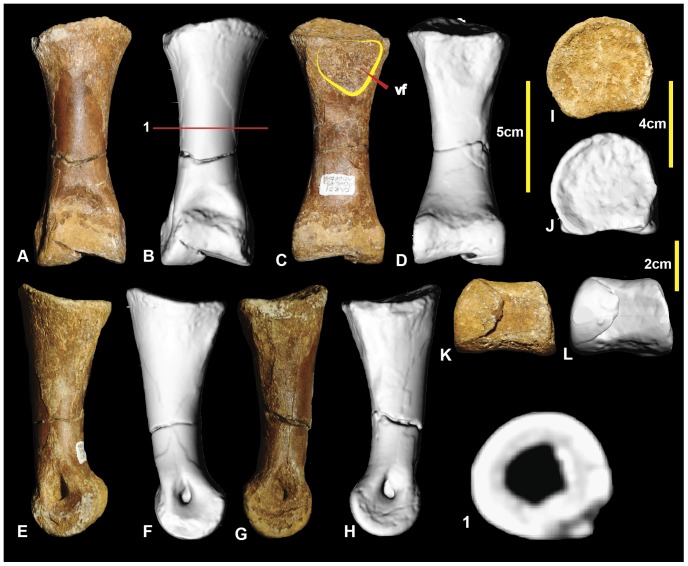
Right pedal phalanx III-2. Right pedal phalanx III-2 in: dorsal (A & B); ventral (C & D); medial (E & F); lateral (G & H); proximal (I & J); and distal (K & L) views; and right pedal phalanx III-2 mid-shaft cross-section (1). Abbreviation: vf, ventral facet.

### Right and left pedal phalanges III-3 (Figure 17; Figure S17)

Right pedal phalanx III-3 is complete. The shaft is subcircular in mid-section (Figure 17.1). A well-defined hyperextensor pit is located on the dorsal surface immediately proximal to the distal condyles. A shallow depression is located at the proximal end of the ventral surface. A groove originates on the medial surface terminates on the ventrodistally emphasizing the ventral heel. The ventral heel terminates around mid-length of the phalanx. The proximal articular surface has two articular facets. The medial articular facet is taller and narrower relative to the broader lateral facet. The distolateral condyle is slightly taller than the distomedial condyle. Both condyles possess deep, well-defined collateral ligament pits. The left pedal phalanx is poorly preserved, with only a very fine veneer of bone preserved distal to the articular surface. In proximal aspect, the articular surface reveals the exact height of the proximal end, which was not preserved in the right specimen. Measurements in Table 3.

**Figure 17:**
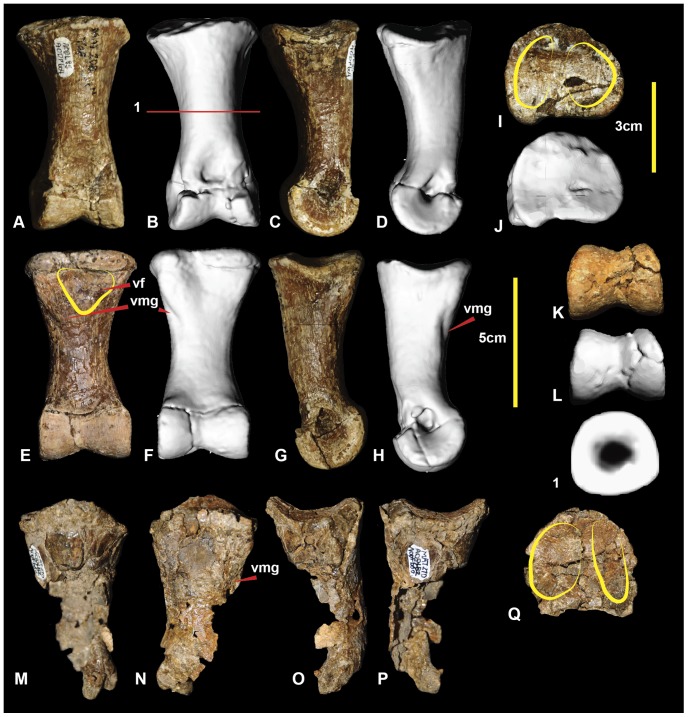
Right and left pedal phalanges III-3. Right pedal phalanx III-3 in: dorsal (A & B); lateral (C & D); ventral (E & F); medial (G & H); proximal (I & J); and distal (K & L) views; and right pedal phalanx III-3 cross-section (1). Left pedal phalanx in: dorsal (M); ventral (N); lateral (O); medial (P); and proximal (Q) views.

### Left pedal phalanx III-4 (Figure 18; Figure S18)

**Figure 18:**
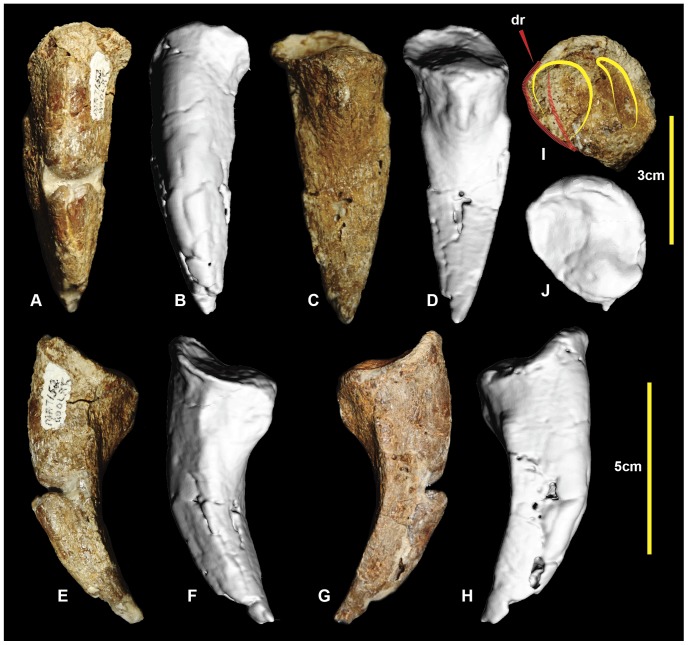
Left pedal phalanx III-4. Left pedal phalanx III-4 in: dorsal (A & B); ventral (C & D); lateral (E & F); medial (G & H); and proximal (I & J) views; and pedal phalanx III-4 cross-section (1).

Pedal phalanx III-4 is a nearly complete ungual. It is recurved laterodistally and tapers along its length to a sharp point. A prominent ridge extends along the medial edge and tapers distally to a point. The lateral ridge is less prominent than this ridge in phalanx II-3 and unlike that of phalanx I-2. A distinct, pinched tubercle is present proximally on the ventral surface of phalanx III-4. The lateral rim of the proximal articular surface bears a distinct rugose growth extending from the lateral articular facet surface. This rugosity is unusual and has not been observed in any of the other unguals of *Australovenator*. Therefore, this structure may be pathological in nature, perhaps a result of infection or arthritis surrounding the articular facet of the ungual phalanx. The shape of the proximal articular facet has been distorted by the bony growth. The distal end of left pedal phalanx-III-3 was not preserved to compare the potentially pathological right pedal phalanx III-4 confirm whether a corresponding pathology might have been present there (Figure 18). Measurements in Table 3.

### Left pedal phalanx IV-1 (Figure 19; Figure S19)

**Figure 19:**
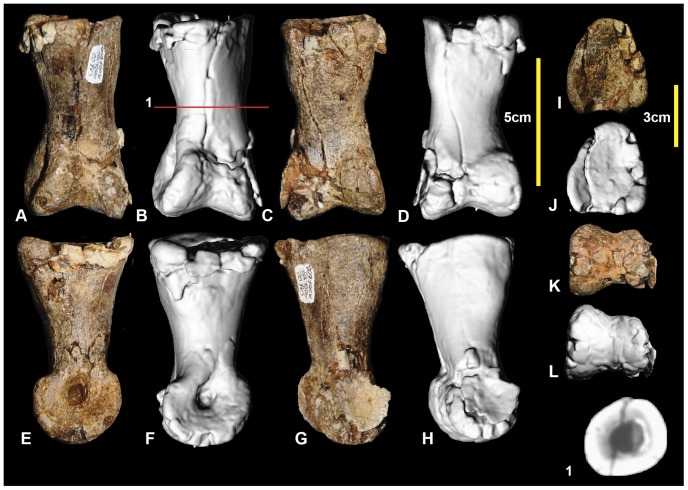
Left pedal phalanx IV-1. Left pedal phalanx IV-1 in: dorsal (A & B); ventral (C & D); medial (E & F); lateral (G & H); proximal (I & J); and distal (K & L) views; and left pedal phalanx IV-1 cross-section (1).

One representative of each of the phalanges of the fourth pedal digit is preserved. Pedal phalanx IV-1 is complete. The proximal articular surface is tall, with a rounded, subtriangular outline in proximal view. The cross-section becomes circular at its mid-length (Figure 19.1). The proximolateral articular facet is angled more laterally than medial facet. The medial and lateral articular facets on the proximal articular surface are not distinctly separated; however, a faint, dorsoventrally oriented ridge weakly divides them (Figure 19J). The medial distal condyle has a deep collateral ligament pit whereas the lateral collateral ligament is relatively shallow and obscured by matrix. The medial condyle is distinctly taller and distomedially splayed compared to the lateral condyle which is angled laterodistally. Measurements in Table 3.

### Left pedal phalanx IV-2 (Figure 20; Figure S20)

Pedal phalanx IV-2 is a poorly preserved specimen with both proximal and distal ends incomplete. In proximal view, the lateral articular facet appears broader than the medial. The shaft has a rounded, sub-trapezoidal cross-section at its mid-length. The medial distal condyle is taller and posteroventrally oriented compared to the lateral condyle, which is splayed laterally. The medial condyle has a deep collateral pit preserved. The ventral heel consists of a ventromedial and ventrolateral processes. The lateral process is slightly more bulbous than the medial. Measurements in Table 3.

### Left pedal phalanx IV-3 (Figure 21; Figure S21)

Pedal phalanx IV-3 is near complete. The proximal articular surface is triangular. The lateral articular facet is slightly broader than the medial facet. The medial distal condyle is taller and slightly broader than the lateral distal condyle. Both medial and lateral condyles have deep collateral ligament pits. The lateral surface of the phalanx is slightly concave dorsoventrally. This can be seen in the mid-shaft cross-section (Figure 21.1). Measurements in Table 3.

**Figure 20:**
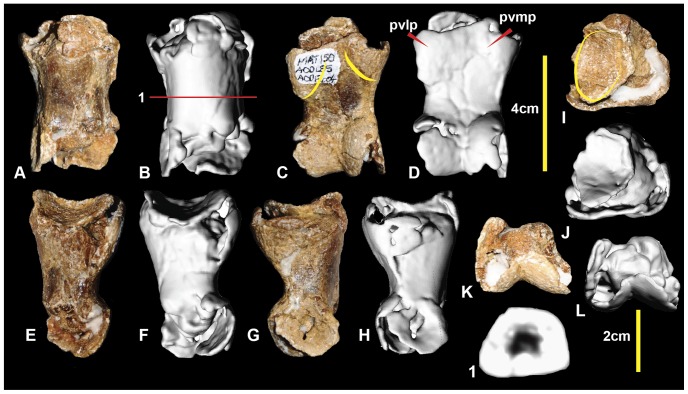
Left pedal phalanx IV-2. Left pedal phalanx IV-2 in: dorsal (A & B); ventral (C & D); lateral (E & F); medial (G & H); proximal (I & J); and distal (K & L) views; and left pedal phalanx IV-2 cross-section (1). Abbreviations: pvlp, proximal ventral lateral process; pvmp, proximal ventral medial process.

**Figure 21:**
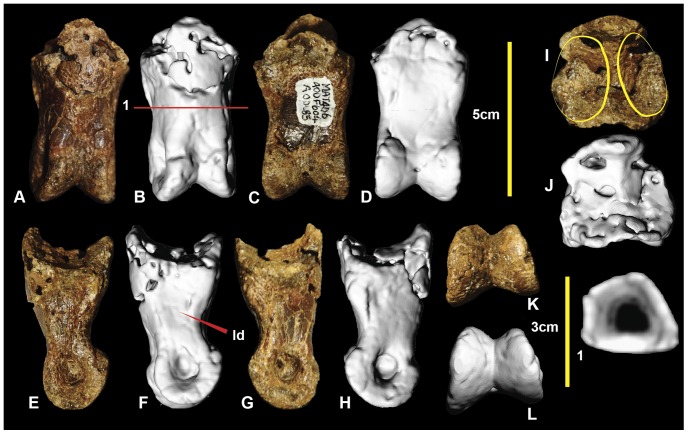
Left pedal phalanx IV-3. Left pedal phalanx IV-3 in: dorsal (A & B); ventral (C & D); lateral (E & F); medial (G & H); proximal (I & J); and distal (K & L) views; and left pedal phalanx IV-3 cross-section (1). Abbreviation: ld, lateral depression.

### Right pedal phalanx IV-4 (Figure 22; Figure S22)

Pedal phalanx IV-4 is complete. The proximal articular surface is subtriangular in proximal view. It bears paired medial and lateral facts. The medial facet is proportionally taller and narrower than the lateral facet. A prominent proximomedial eminence is present on the ventral surface. The medial surface has prominent ridge that originates from the medial articular facet rim and tapers into the ventral portion of the medial condyle. The ridge is bounded on its dorsal and ventral sides by shallow depressions the ventral larger than the dorsal. The lateral distal condyle is slightly broader, but shorter than the medial distal condyle. Measurements in Table 3.

### Right pedal phalanx IV-5 (Figure 23; Figure S23)

Pedal phalanx IV-5 is a complete ungual phalanx. It is recurved and tapers distally to a distinct point. It possesses near symmetrical medial and lateral grooves. In ventral view, the ungual is spear-shaped. The lateral leading edge is slightly longer than the medial side due to weak distomedial curvature. There is a distinct ventrally pinched flexor tubercle at the proximal end of the ventral surface. The medial articular facet is angled medially in comparison to the lateral facet. Measurements in Table 3.

### Reconstructed metatarsus and pes (Figure 24, 25, Figure S24)

A near complete right pes has been reconstructed using specimens from the right pes and mirrored elements only known from the left pes. Unfortunately pedal phalanx I-1 is unknown. Despite this, the pes is the most complete amongst neovenatorids.

### Neovenatorid comparisons

Neovenatoridae was established [20] on the basis of synapomorphies shared among Neovenator salerii [13], Aerosteon riocoloradensis [21], Chilantaisaurus tashuikouensis [22], Fukuiraptor kitadaniensis [23], Megaraptor namunhuaiquii [24], Orkoraptor burkei [25] and Australovenator wintonensis [1]. Recently, a comparison of the forearm of Australovenator was made with each of these taxa where possible [11]. Herein, we compare hind limb elements of Australovenator with these neovenatorid taxa for future cladistic analysis. Published illustrations and descriptions were used to make comparisons. Because not all specimens were examined directly, some comparisons are incomplete.

**Figure 22:**
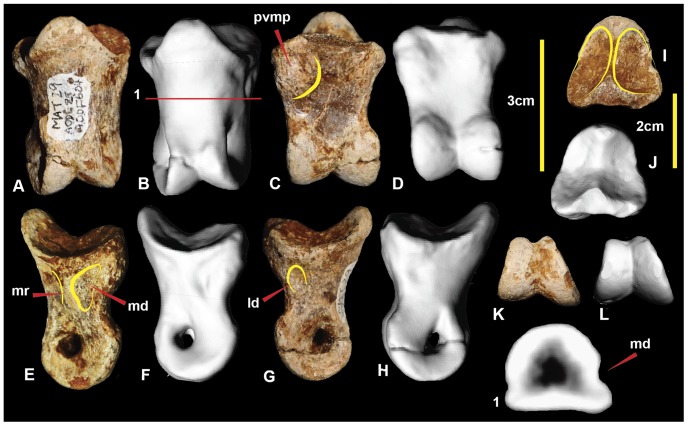
Right pedal phalanx IV-4. Right pedal phalanx IV-4 in: dorsal (A & B); ventral (C & D); medial (E & F); lateral (G & H); proximal (I & J); and distal (K & L) views; and right pedal phalanx IV-4 cross-section (1). Abbreviations: ld, lateral depression; md, medial depression; mr, medial ridge; pvmp, proximal ventral medial process.

**Figure 23:**
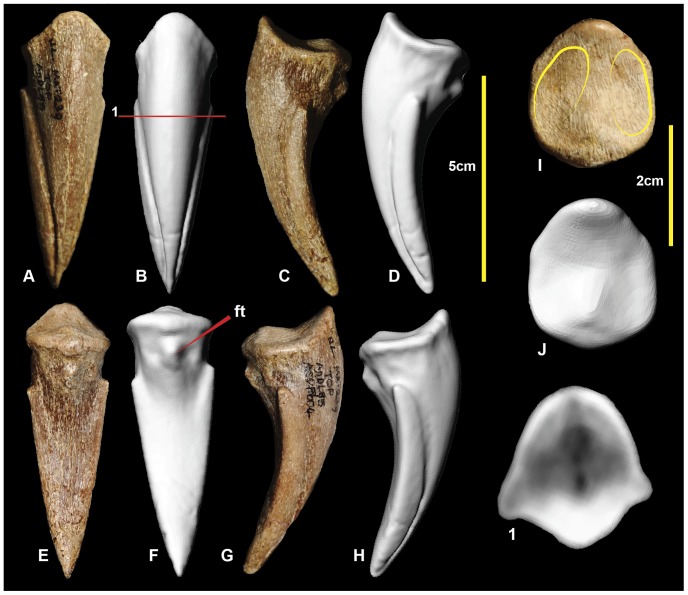
Right pedal phalanx IV-5. Right pedal phalanx IV-5 in: dorsal (A & B); medial (C & D); ventral (E & F); lateral (G & H); and proximal (I & J) views; and right pedal phalanx IV-5 cross-section (1). Abbreviation: ft, flexor tubercle.

**Figure 24:**
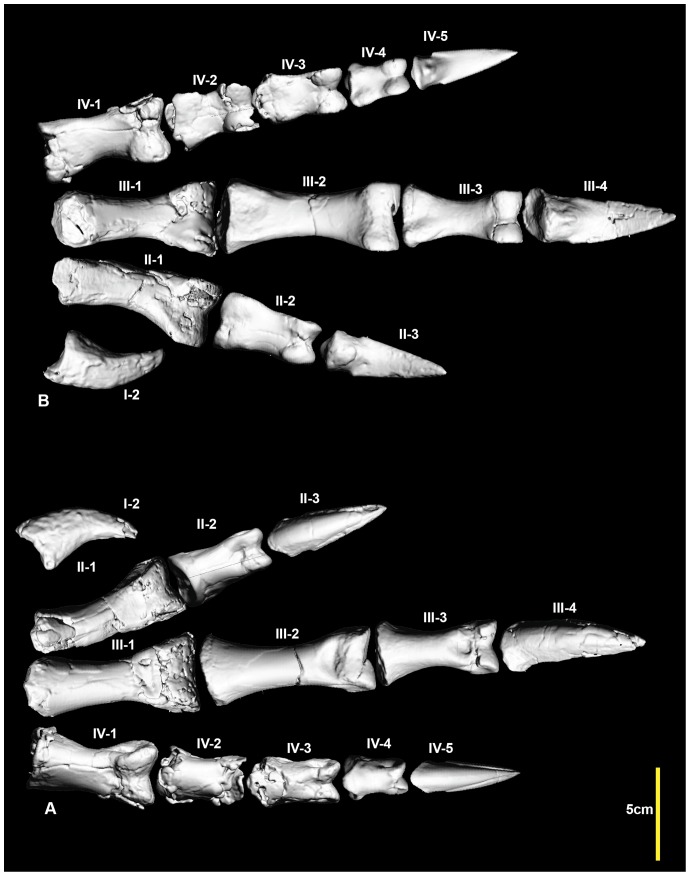
Reconstructed right pes. Reconstructed right pes in: dorsal (A); and ventral (B) views.

**Figure 25:**
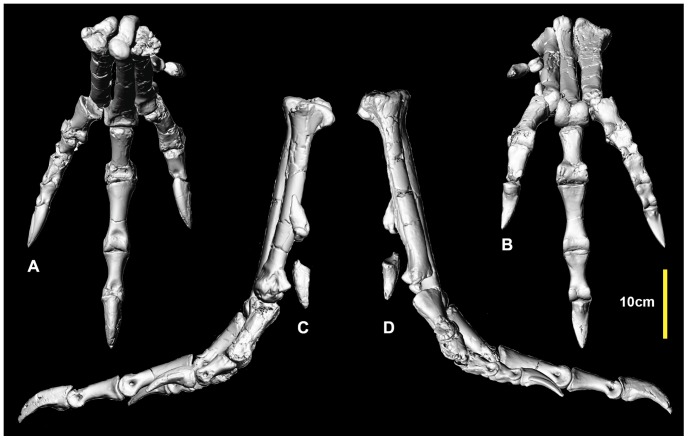
Reconstructed right metatarsus and pes. Reconstructed right metatarsus and pes in: dorsal (A); ventral (B); medial (C) and lateral (D) views.

### Femur

*Australovenator, Neovenator*, *Fukuiraptor* and *Chilantaisaurus* have preserved femora. *Australovenator* and *Fukuiraptor* have a groove visible in proximal view that tapers disto-laterally on the caudal surface. The groove is bounded by a pronounced flange which is labelled the posterior flange of the caput in Brusatte (Figure 21B in [18]). This groove and flange are less prominent in *Neovenator* and *Chilantaisaurus*. *Australovenator* and *Fukuiraptor* differ in that this flange buttresses the femoral head medially in *Fukuiraptor,* whereas it curves caudally around the femoral head in *Australovenator* (Figure 2IJ); (Figure 12C in [23]); and (Figure 21E in [18]). However, we note the high level of morphological variability among specimens of *Fukuiraptor* [23].

The outline of the femur in distal view is similar in *Australovenator* and *Neovenator*, in which the medial and lateral condyles have a bulbous appearance, resulting in a narrower flexor groove and a shallow extensor groove (this is autapomorphically shallow and narrow in *Neovenator* (Figure 21 in [18]). The femora of *Fukuiraptor* and *Chilantaisaurus* have widely spaced distal condyles, creating a much larger flexor groove and extensor grooves (see Figure 2KL; Figure 12D in [23]; Figure 21F in [18]; Figure 4FG in [26]). The cranial surface of the femoral shaft of *Neovenator* bears a distinct, cranial intermuscular line (Figure 21A in [18]). This is not visible in *Australovenator* or *Chilantaisaurus*, perhaps due to poor preservation or less advanced ossification, and was not illustrated in *Fukuiraptor* [23]. The position of the fourth trochanter and its associated muscle scar are similar in *Australovenator* (Figure 2CD) and *Neovenator* (Figure 21B-D in [18]), whereas it is more proximally positioned in *Fukuiraptor* (Figure 12A in [23]). The crista tibiofibularis is similar in both *Neovenator* (Figure 21BC in [18]) and *Australovenator* (Figure 2CD). It is difficult to identify the shape of this process in *Fukuiraptor* and *Chilantaisaurus*, because of damage. *Australovenator, Fukuiraptor and Neovenator* possess a distally projecting medial condyle which is a morphological trait used in defining the Neovenatoridae clade, that also appears in some carcharodontosaurids [20].

### Tibia

*Australovenator, Neovenator Fukuiraptor, Orkoraptor* and *Chilantaisaurus* all preserve at least partial tibiae. In proximal view, the tibial head of *Australovenator* is closer in morphology to *Fukuiraptor*. The tibial head in both *Neovenator* (Figure 22E in [18]) and *Orkoraptor* (Figure 7C in [25]) in proximal view has the medial condyle more caudally positioned in than in *Australovenator* (Figure 3IJ) and *Fukuiraptor* (Figure 13C in [23]). The lateral condyle of proximal femur bears a spine-like anteroventral process in *Australovenator* (Figure 3B) and *Fukuiraptor* (Figure 13E in [23]) whereas in *Neovenator* (Figure 22C in [18]) it forms a sharp hook. This feature is not preserved in *Chilantaisaurus.* In distal view there do not appear to be major differences between *Neovenator*, *Chilantaisaurus* and *Australovenator*; the distal end of the tibia was not preserved in *Fukuiraptor*.

### Fibula

Complete fibulae are known for *Australovenator* (Figure 4) and *Neovenator* (Figure 23 in [18]) and a partial fibula of *Chilantaisaurus* is also known. The preservation of *Chilantaisaurus* does not allow meaningful morphological comparisons to be made. The majority of the *Australovenator* shaft is more elongate and slender than *Neovenator*.

### Astragalus

Astragali are known in *Fukuiraptor* (Figure 14 in [23]) and *Australovenator* (Figure 5). There is a distinct dorsoventrally oriented ridge on the caudal surface that curves proximally from a central position to a lateral position in *Australovenator* which does not appear to be present in *Fukuiraptor* (Figure 14C in [23]). In cranial view, the proximal cranial groove appears shallowly concave in *Fukuiraptor* whereas it has a distinct distal groove on the lateral side of the *Australovenator* specimen.

### Metatarsus

**Figure 26:**
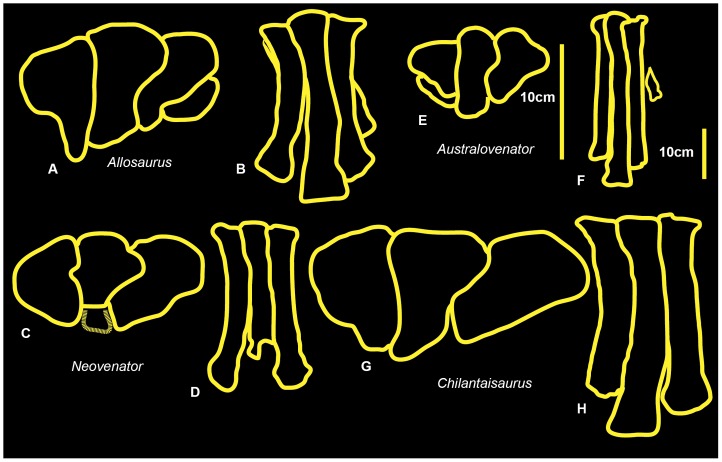
Right metatarsus comparisons. *Allosaurus* in: proximal (A); and dorsal (B) views. *Neovenator* in: proximal (C) and dorsal (D) views. *Australovenator* in: proximal (E) and dorsal (F). *Chilantaisaurus* in: proximal (G) and dorsal (H).

Comparisons were made of metatarsi from the primitive (and geologically older) to derived (and geologically younger; although *Chilantaisaurus* is slightly younger than *Australovenator*) allosauroid theropods *Allosaurus* (Late Jurassic, U.S.A), *Neovenator* (Early Cretaceous, United Kingdom), *Chilantaisaurus* (early Late Cretaceous, China), *Megaraptor* (early Late Cretaceous, Argentina) and *Australovenator* (Upper Cretaceous Australia) (Figure 26). The proximal surface of metatarsal II of *Australovenator* was not preserved however the medial margin of metatarsal III was straight implying that the lateral margin of metatarsal II was also straight. This straight feature was also recognised in *Fukuiraptor* (Figure 16F in [23]).

This margin is slightly bulbous in both *Neovenator* (Plate 42, 5 in [18]) and *Chilantaisaurus* (Figure 6B in [26]), however *Neovenator* has a slight medial groove, which is absent in *Chilantaisaurus*. In proximal view, metatarsal III is bulbous dorsally on the lateral margin in *Australovenator*. This feature is shared with *Neovenator* (Plate 44, 3 in [18]). In *Chilantaisaurus* (Figure 6B in [26]) this bulbous feature is reduced, creating a slightly curved lateral margin. Unfortunately this feature is too hard to distinguish in *Fukuiraptor* (Figure 16L in [23]). The morphology of the distal surface of metatarsal III of *Megaraptor* (Figure 2 in [24]) is nearly identical to that of *Australovenator*. The proximal end of metatarsal IV of *Australovenator* has a rounded, subtriangular outline, with a concave medial margin buttressing the bulbous dorsal end of metatarsal III. Metatarsal IV of *Megaraptor* (Figure 10B in [27]) shares the closest morphology to *Australovenator* with the same concave medial margin to buttress the bulbous end of metatarsal III. *Neovenator* also shares this concavity, though the lateral margin of this element is more rounded and oval (Plates 44, 3 in [18]). Metatarsal IV of *Chilantaisaurus* lacks this concave medial margin and is trapezoidal (Figure 6B in [26]).

### Pedal phalanges

Comparison of neovenatorid pedal phalanges is difficult if recovered specimens do not directly articulate. It is therefore difficult to accurately compare the *Fukuiraptor* specimens (Figure 18 in [23]) with *Australovenator*. The only other neovenatorid currently represented by articulated pedal phalanges is *Neovenator*, which facilitated the following comparisons. In some cases the poor preservation of *Australovenator* specimens meant that meaningful comparisons were not achievable.

### Pedal phalanx II-1

In cranial view, pedal phalanx II-1 is elongated and distinctly bows medially in *Australovenator*, whereas it is relatively straight and more robust in *Neovenator* (Table 4). The *Australovenator* specimen also appears proportionally narrower at its proximal end compared to the wider proximal end of *Neovenator* (Plate 44, 1 in [18]). In medial and lateral views, the distal condyles of *Neovenator* (Plates 44, 4 and 45, 2 in [18]) are pronounced more caudally with respect to the shaft of the phalanx than in *Australovenator*. The proximo-ventral articulation of *Neoventor* is also more ventrally pronounced on the phalanx than in *Australovenator*. The proximal surface of the *Australovenator* specimen is proportionally taller compared to that of *Neovenator* (Table 4).

**Table 4:** Pedal phalanx ratio comparisons. Measurements obtained [18].

Dinosaur	Length	Mid-shaft width	Width/length	Proximal height	Proximal width	Width/height
***Neovenator*** ** MTII-1**	109	38	0.35	60	62	1.03
***Australovenator*** ** MTII-1**	106*	28	0.26	43	33	0.76
***Neovenator*** ** MTII-2**	88	34	0.38	52	37	0.7
***Australovenator*** ** MTII-2**	64*	26	0.41	30*	35*	1.16
***Neovenator*** ** MTIV-1**	83	38	0.46	56	54	0.96
***Australovenator*** ** MTIV-1**	82	27	0.33	46	39	0.85
***Neovenator*** ** MTIV-2**	69	35	0.5	41	46	1.12
***Australovenator*** ** MTIV-2**	49	25	0.51	30*	35*	1.16
***Neovenator*** ** MTIV-3**	34	37	1.08	34	37	1.08
***Australovenator*** ** MTIV-3**	33	21	0.63	26	28	1.07
***Neovenator*** ** MTIV-4**	34	37	1.08	34	37	1.08
**Australovenator MTIV-4**	33	21	0.63	26	28	1.07

Length, width and proximal height comparisons of *Australovenator* and *Neovenator*. The ratios reveal that *Australovenator* possessed a more elongate pes. Asterisks (*) mark lengths which have been estimated due to poor preservation.

### Pedal phalanx III-4

In ventral aspect, pedal phalanx III-4 has a distinct pinched flexor tubercle; this is less pronounced in *Neovenator* (Plate 45, 3 in [18]) than in *Australovenator*.

### Pedal phalanx IV-1

The *Australovenator* specimen curves distolaterally along its length whereas the *Neovenator* specimen appears relatively straight. The *Neovenator* specimen (Plates 44, 1 and 45, 8 in [18]) also has a much blockier proximal end than the proportionally taller *Australovenator* specimen (Table 4).

### Pedal phalanx IV-3

The pedal phalanx IV-3 of *Australovenator* is more elongate than that of *Neovenator*. The *Neovenator* specimen has a very short shaft which is almost indistinguishable from the proximal and distal ends (Plates 44, 9 in [18]) (Table 4).

### Pedal phalanx IV-4

The pedal phalanx IV-4 of *Australovenator* is proportionally narrower and more elongate than *Neovenator*. It has a small section of phalanx shaft visible between the distal condyles and proximal articular facet whereas the shaft is indistinguishable in *Neovenator.* The *Australovenator* specimen also appears proportionally taller in proximal aspect (Table 4).

### Hind limb element proportions (Table 2)

The hind limb element proportions of *Australovenator* were compared with *Neovenator*, *Chilantaisaurus, Fukuiraptor* and *Allosaurus*. The *Fukuiraptor* specimen measurements from the holotype description were used for this analysis. Unfortuneatly the tibia is incomplete but it was referred to as being a similar length to the femur [23]. As this is not an exact measurement the percentage result should be used with caution. To achieve a comparable limb proportion with *Fukuiraptor* we calculated a metatarsus to femur proportion percentage. Interestingly this proportion indicates that *Fukuiraptor* has the most elongate metatarsus to femur length next to *Australovenator*. The proportions indicate a larger body plan is supported with a wider but shorter metatarsus.

*Neovenator* is the only other neovenatorid theropod in which the positions of the pedal phalanges have been determined. A comparison of their dimensions (height, width and length) with those of *Australovenator* indicated that the pedal phalanges of *Australovenator* are more elongate than *Neovenator* (Table 4).

## Conclusion

These newly described hind limb elements of *Australovenator*, together with the holotype specimens previously described, mean that the hind limb of *Australovenator* is the most complete of any neovenatorid known to date. The discovery of the new hind limb elements enabled exact skeletal positions to be determined for each of the holotype pedal phalanges, as well as those newly described here. This will provide a point of comparison for future neovenatorid pedal phalanges and should ensure more accurate determination of pedal position of isolated phalanges. The morphology of the metatarsus was found to be similar in *Australovenator* and *Megaraptor*, as demonstrated by metatarsals III and IV. Comparisons of *Australovenator* specimens with published figures and measurements of, *Neovenator, Fukuiraptor* and *Chilantaisaurus* revealed that in relation to body size *Australovenator* had the most elongate hind limb and stride length. Additionally the hind limb proportions indicate that larger forms possessed a shorter but wider metatarsus in comparison to proportionally smaller neovenatorids.

The morphological descriptions provided here are supplemented with two- and three-dimensional figures. The 3-D figures will allow other researchers to more accurately observe the hind limb elements of *Australovenator* than would otherwise be possible in a locality remote from the AAOD museum in which the specimens are housed.

## Supporting Information

Figure S1 Site photographs
(PDF)

Figure S2 Femur
(PDF)

Figure S3 Tibia
(PDF)

Figure S4 Fibula
(PDF)

Figure S5 Astragalus
(PDF)

Figure S6 Metatarsal I
(PDF)

Figure S7 Metatarsal II
(PDF)

Figure S8 Metatarsal III
(PDF)

Figure S9 Metatarsal IV
(PDF)

Figure S10 Reconstructed metatarsus
(PDF)

Figure S11 Pedal phalanax I-2
(PDF)

Figure S12 Pedal phalanx II-1
(PDF)

Figure S13 Pedal phalanx II-2
(PDF)

Figure S14 Pedal phalanx II-3
(PDF)

Figure S15 Pedal phalanx III-1
(PDF)

Figure S16 Pedal phalanx III-2
(PDF)

Figure S17 Pedal phalanx III-3
(PDF)

Figure S18 Pedal phalanx III-4
(PDF)

Figure S19 Pedal phalanx IV-1
(PDF)

Figure S20 Pedal phalanx IV-2
(PDF)

Figure S21 Pedal phalanx IV-3
(PDF)

Figure S22 Pedal phalanx IV-4
(PDF)

Figure S23 Pedal phalanx IV-5
(PDF)

Figure S24 Reconstructed metatarsus and pes.
(PDF)
