# Supplementary figures and images for: New Australovenator Hind Limb Elements Pertaining to the Holotype Reveal the Most Complete Neovenatorid Leg
Source: PLoS One. 2013 Jul 24;8(7):e68649. doi: 10.1371/journal.pone.0068649 (PMC3722220; doi:10.1371/journal.pone.0068649)

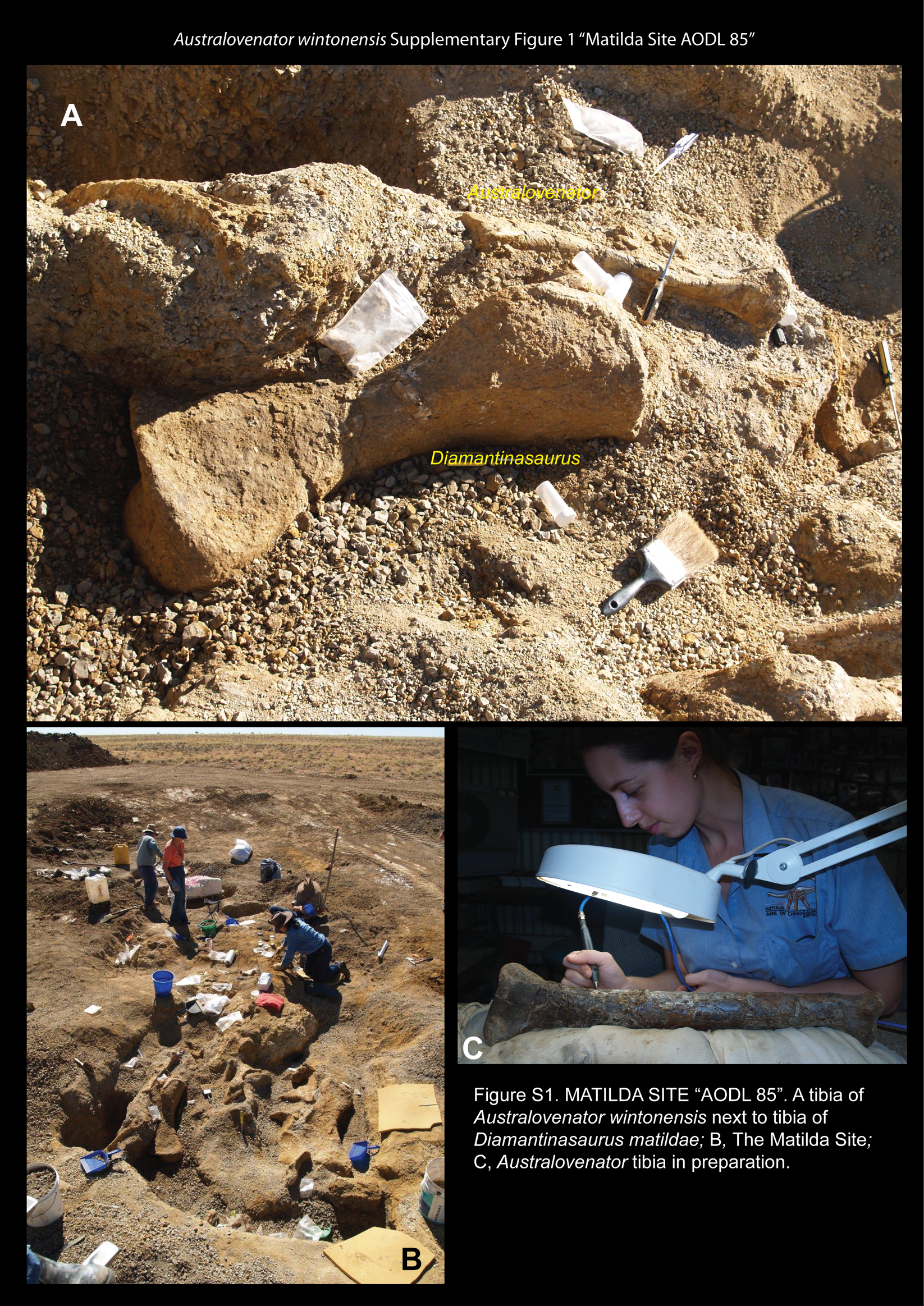

Supplement: Figure S1 — Site photographs (PDF) [file pone.0068649.s001.pdf]
