# Supplementary material for: New Australovenator Hind Limb Elements Pertaining to the Holotype Reveal the Most Complete Neovenatorid Leg
Source: PLoS One. 2013 Jul 24;8(7):e68649. doi: 10.1371/journal.pone.0068649 (PMC3722220; doi:10.1371/journal.pone.0068649)
Supplement: Figure S10 — Reconstructed metatarsus (PDF) [file pone.0068649.s010.pdf]

***Australovenator wintonensis* Supplementary Figure 10 Right Metatarsus**

Australovenator specimens were discovered by and are housed in The Australian Age of Dinosaurs Museum of Natural History. Access to the specimens was granted by founder and chairman David A. Elliott. Computed tomography (CT) scanning: Sarah Wooldridge (Queensland Xray Mackay). Model construction: Matt A. White (University of Newcastle) and Travis R. Tischler (AAOD). Visualisation: David G. Barnes (Monash University).
