# Supplementary material for: New Australovenator Hind Limb Elements Pertaining to the Holotype Reveal the Most Complete Neovenatorid Leg
Source: PLoS One. 2013 Jul 24;8(7):e68649. doi: 10.1371/journal.pone.0068649 (PMC3722220; doi:10.1371/journal.pone.0068649)
Supplement: Figure S22 — Pedal phalanx IV-4 (PDF) [file pone.0068649.s022.pdf]

Australovenator wintonensis Supplementary Figure 22 Right Pedal Phalanx IV-4

Australovenator specimens were discovered by and are housed in The Australian Age of Dinosaurs Museum of Natural History. Access to the specimens was granted by founder and chairman David A. Elliott. Computed tomography (CT) scanning: Sarah Wooldridge (Queensland Xray Mackay). Model construction: Matt A. White (University of Newcastle). Visualisation: David G. Barnes (Monash University).
